# Children First

**Published:** 1919-06

**Authors:** 


### "Children First."

"The results of underfeeding or indiscriminate food substitution in childhood are startlingly shown abroad as a result of the war, and are beginning to be evident in our own great cities." And "milk has no substitute in the diet of the child." These and other unqualified statements of the importance of guarding the milk supply to prevent the physical deterioration of American children during the war are scattered through the latest report issued by the Children's Bureau of the U. S. Department of Labor and entitled "Milk, the Indispensible Food for Children."

This report, with its striking figures showing a decrease in the amount of milk now available and in the amount which is finding its way to the children in poor homes, has special interest in connection with the campaign to save 100,000 lives of babies and little children during the second year of the war, It not only emphasizes the fact that children who are deprived of milk can not thrive properly, but it analyzes the changes in the production and export of dairy products during the war and shows the necessity of public action.

"The nourishment of our children is the first duty of the Nation. Since milk and milk products are a vital necessity for children, for nursing mothers, and for the sick and wounded, the public should be made to realize that the children's need for dairy products should be assured."

England and Italy have regulated the sale of cream and curtailed the use of butter, in order that their child population might receive the more adequate and economical nourishment offered by whole milk. Germany, early in the war, provided that the adult civilian population might have milk only after the needs of children, mothers, invalids, and the army were met.

The report discusses the various forms in which cows' milk may be used for children. For the young baby, it says, there is nothing so good as mother's milk.

"Never before in the history of civilization has it been so urgent a matter that every child should have breast milk for as long a time as possible, in order that every child that survives birth may have the best chance for life and health."

But for children under 2 other than those breast fed, and for older children, the report states that cows' milk is an absolute necessity if disease and death are to be kept within bounds and if the coming generation is to survive and to sustain the national standards. " 'Children first' should be part of the national food slogan."

"It is the duty now of every individual community to see that its children have milk of good quality and in sufficient amount to assure their normal development. To do this the price of milk must be controlled or fixed, and the milk supply to infants and children carefully safeguarded. The malnutrition of our children was, even before 1914, a serious national problem and one demanding urgent attention. Poverty and ignorance of dietary essentials have been ever-present factors in the malnutrition of the young, and war conditions can not fail to increase the gravity of the situation and the difficulties of maintaining the health of the Nation."

